# Systematic Review of Nonmedical Costs of Firearm Injury

**DOI:** 10.1016/j.amepre.2026.108276

**Published:** 2026-01-21

**Authors:** David W. Hutton, Taylor W. Lefler, Danwei Yang, Shiying Mai, Michael Holtz, Hanwen Zhang, Honey S. Modi, William B. Hillegass, Marc A. Zimmerman, Patrick M. Carter

**Affiliations:** 1 Department of Health Management and Policy, University of Michigan, Ann Arbor, Michigan;; 2 University of Mississippi Medical Center, Jackson, Mississippi;; 3 Department of Public Health Sciences, University of Chicago, Chicago, Illinois;; 4 Division of Social and Administrative Sciences, School of Pharmacy, University of Wisconsin-Madison, Madison, Wisconsin;; 5 Institute for Firearm Injury Prevention, University of Michigan, Ann Arbor, Michigan;; 6 College of Pharmacy, University of Texas at Austin, Austin, Texas;; 7 Stryker, Kalamazoo, Michigan;; 8 Department of Health Behavior & Health Equity, University of Michigan, Ann Arbor, Michigan;; 9 Department of Emergency Medicine, University of Michigan, Ann Arbor, Michigan

## Abstract

**Introduction:**

To evaluate the impact of firearm injury prevention programs and policies, it is important to characterize firearm injury costs. A prior review evaluated medical-specific firearm injury costs, but nonmedical societal costs have not been previously reviewed. This study explicitly reviews the nonmedical costs of both fatal and nonfatal firearm injury.

**Methods:**

A systematic review of studies conducted from 2000 to 2023 in English reporting nonmedical costs of U.S. firearm injury in Embase, PubMed, the Cochrane Library, EconLit, JSTOR, and gray literature was performed. The methods, data quality, types of nonmedical costs, and the relationship between nonmedical and medical costs were extracted. Bias was assessed using a modified Newcastle-Ottawa Scale and synthesized on the basis of the SWiM (Synthesis Without Meta-analysis) guidelines.

**Results:**

Nineteen studies analyzing national, state, city, and individual costs were identified. Studies generally used modeling approaches (13 studies) to calculate costs, but 4 used a cohort approach, and 2 used a willingness-to-pay approach. Studies evaluated wide ranges of costs: medical, productivity, intangible (quality of life), criminal justice, and other costs. In studies evaluating both medical and nonmedical costs, nonmedical costs were much higher, with criminal justice costs being 1.5−3.9 times larger, productivity costs being 14−25 times larger, and intangible costs being 29−175 times larger than medical costs.

**Discussion:**

The literature on nonmedical costs is relatively underdeveloped, leading to wide ranges in results. Studies consistently show that nonmedical costs are in orders of magnitude larger than medical costs of firearm injury with total costs potentially in the millions of dollars per injury. More research on nonmedical costs of firearm injury will help quantify and clarify the magnitude of these costs as well as be used to understand the cost-savings of specific prevention policies or programs.

## INTRODUCTION

An estimated 44,446 people died from firearms in 2024.^1^ About 27,592 of those deaths were suicides; 15,364 were homicides, with the rest being unintentional, legal intervention, and undetermined. In 2019, the federal government allocated specific funding for firearm injury prevention research^2^; however, cuts to the Centers for Disease Control and Prevention may put that funding in jeopardy.^3,4^ To understand the value of reducing firearm injuries, it is important to understand the economic impact of firearm injuries. This cost can be multifaceted: medical costs, criminal justice costs, productivity costs, and more subjective costs related to quality of life or feelings or perceptions of safety.

A 2024 systematic review of medical costs of firearm injury found that costs ranged from $281 to $570,619, with a median cost per injury of $29,974 (inflation adjusted to 2025).^5^ This review also suggests that these estimates may be underestimates because most studies excluded costs after initial acute care hospitalizations. Although this study focused on important burdens of medical costs, it did not include other costs to society, such as criminal justice or productivity, which could be very large. The review presented in this paper specifically examines nonmedical costs of both fatal and nonfatal firearm injury to gain a more complete picture of the overall societal costs of firearm injury.

## METHODS

This research and its predetermined protocol were registered in advance on the International Prospective Register of Systematic Reviews website, under the protocol Identification CRD42023451711. Approval from the IRB was not necessary because the study did not involve human subjects.

Literature searches were conducted from 2000 to July 1, 2023 on Embase, PubMed, the Cochrane Library, EconLit, and JSTOR. Gray literature articles are also included. Search terms were (firearm* OR Gun OR gunshot OR handgun OR shoot*) AND (Violence OR injur* OR psychological OR death OR mortality OR mental) AND (*economic OR cost OR spending OR financial OR expenditure OR productivity*). Studies referenced from these studies and additional studies found in the gray literature were added (e.g., technical reports).

Studies were included if they were published in or after the year 2000, were written in English, and reported nonmedical societal monetary costs of firearm injuries in the U.S. This encompassed unintentional (accidental) injuries, suicide, interpersonal violence, community violence, and intimate partner violence. Studies detailed direct costs, indirect costs, intangible costs, overall costs, and other societal economic consequences attributable to firearm injuries. Studies only including medical costs (outpatient, inpatient, mental health, long-term care, pharmaceuticals) were excluded. Studies not identifying injury costs specifically from firearms were excluded. Costs due to wartime injuries were excluded. Meeting abstracts and commentary were also excluded.

Two investigators independently screened titles and abstracts for a full-text review. A third investigator resolved conflicts. Studies chosen for full-text review had a similar independent evaluation by 2 investigators, with disagreements settled by a third. Covidence, a screening and data extraction software tool for conducting systematic reviews, was used for this process. Researchers also snowballed the reference lists from papers included in the data extraction to ensure no relevant studies were missed.

The outcome evaluated for this study was the societal monetized cost associated with firearm injuries. Two investigators independently extracted data from the full-text papers and reached a consensus. Extracted data included general information (study identification, title, author, country in which the study was conducted, geography), method of included studies (aim of the study, source of unit cost information, source of incidence information, study design, study start/end date, year of inflation adjustment, study funding source, possible conflicts), and participants of included studies (population description, inclusion and exclusion criteria, type of firearm injury, firearm type, method of recruitment of victims, total number of participants). Detailed cost data were extracted and broken out by types of costs as reported in each study.

The quality of the studies and the risk of bias were assessed using a modified Newcastle-Ottawa Scale, which is designed to evaluate the quality of observational studies. This assessment includes several critical questions, including whether the study included a comparison group, whether all study groups were derived from similar source or reference populations, the validity of exposure and outcome measures, whether investigators were blinded to endpoint assessment, and whether potential confounders (such as comorbidities and multi-component interventions) were identified. In addition, the sources of unit cost information and incidence data were recorded. All costs were inflation adjusted to April 2025 values using the gross domestic product (GDP) deflator.

The strategy for data synthesis was based on the SWiM (Synthesis Without Meta-analysis) guidelines. Because studies were anticipated to be heterogeneous, a meta-analysis would not be appropriate. Effect estimates were summarized in tables and graphs. Because the studies were heterogeneous, it is difficult to make direct comparisons among studies. For studies that included similar cost categories, examined similar populations, or employed comparable methodologies, relationships between different cost types within the studies were examined. For studies that included both medical and nonmedical costs, the relative magnitudes of nonmedical to medical costs were compared in a variety of cost categories. Costs of fatal and nonfatal injuries were compared in studies that included both.

## RESULTS

**Figure 1.**
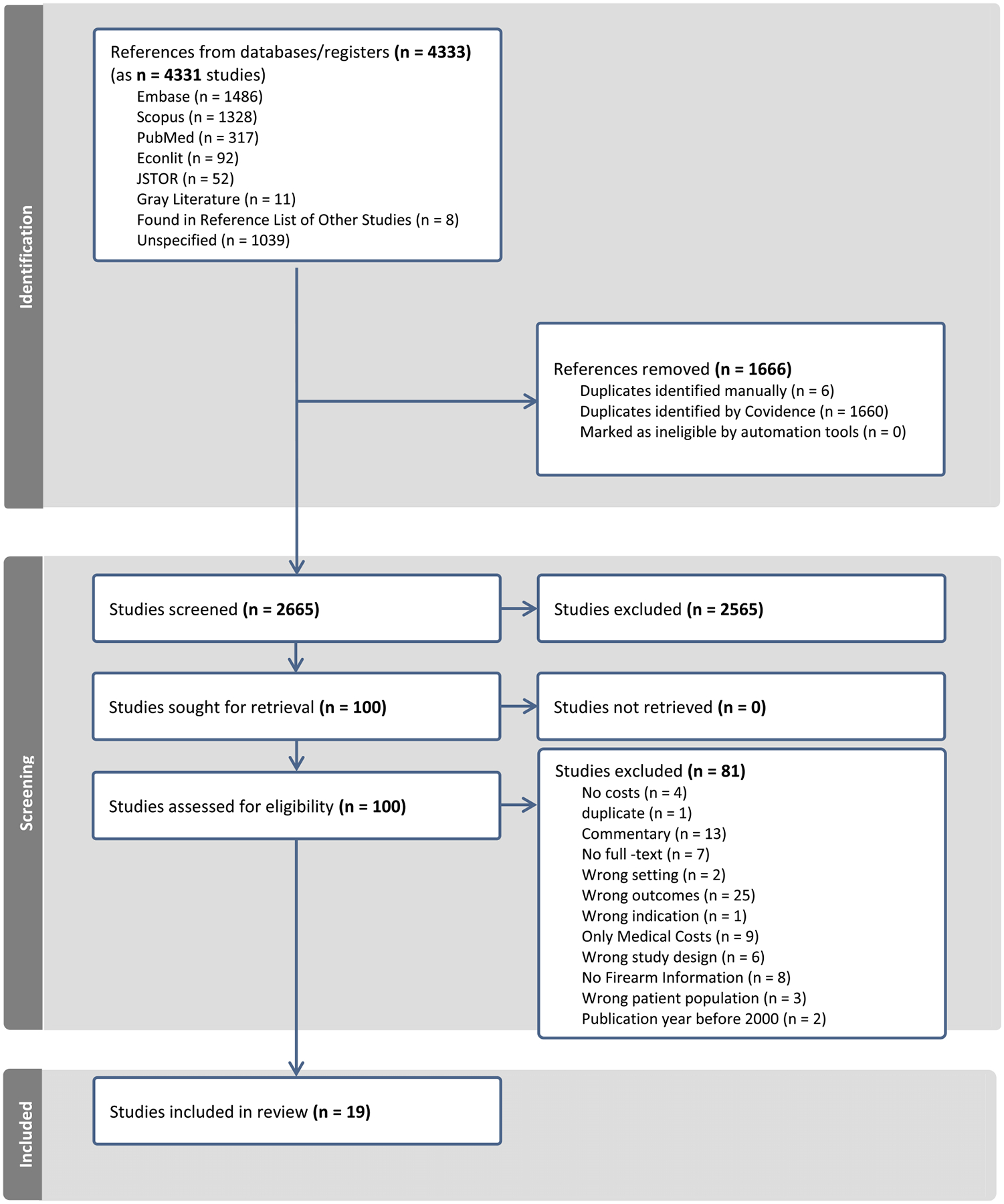
Prisma diagram.

A total of 4,333 potential references were identified, 1,666 of which were duplicates, with a total of 2,665 for screening. Of these, 2,565 were excluded after a title and abstract review, leaving 100 articles for full-text review. Of these, 81 did not meet inclusion criteria (e.g., did not include societal costs), and 19 articles remained for evaluation (Figure 1).

Included studies were published from 2000 to 2023 and identified a variety of potential economic impacts of firearm injury, even if they were unable to quantify all those effects. Appendix Table 1 (available online) shows the cost types and the ways in which they are quantified or discussed.

Among the 19 sources included in this study that analyze societal costs of firearm-related fatalities and injuries, 13 articles utilized modeling approaches to estimate costs associated with firearm injury events, 2 articles focused on estimating the willingness to pay to avoid these events, and 4 articles extracted costs from patients in cohort studies (Appendix Table 2, available online).

Mathematical or statistical models were the most used approaches. Several papers relied on the Centers for Disease Control and Prevention’s Web-based Injury Statistics Query and Reporting System (WISQARS) for identifying national-level injury data and associated costs.^6–9^ Prior to 2015, WISQARS employed a mathematical modeling approach to estimate injury costs, deriving average per-case costs from national surveys and historical data, calculating total costs by multiplying these averages by injury incidence. In 2015, WISQARS transitioned to statistical modeling (e.g., regression) using financial transaction data. This statistical approach accounts for variations in costs on the basis of factors such as injury severity, demographics, and treatment settings. The value of statistical life was used to calculate nonmedical costs for fatal injuries.^8^ Similar to the WISQARS model, National Institute for Criminal Justice Reform,^10^ Everytown^9^ (2022), Peters et al.^11^ (2020), Follman and colleagues,^12^ Corso et al.^13,14^ (2006, 2007), Finkelstein and colleagues^15^ (2006), and Lemaire^16^ (2005) applied fixed average costs to injury counts to estimate total costs, and Irvin-Erickson et al.^17^ (2017) used realworld data and statistical techniques to estimate costs while accounting for cost variability. Many of these studies rely on the work of the researcher Ted Miller and his Pacific Institute for Research and Evaluation model.^6–9,13–15,18,12^ This mathematical model estimates costs of firearm injury, including medical, productivity, criminal justice, and quality-of-life losses.

The willingness-to-pay method accounts for both tangible and intangible costs, including the subjective value people place on safety and concerns for their own and other’s welfare, as well as costs associated with preventing or avoiding firearm injury. Cook and Ludwig (2000)^19^ surveyed individuals’ willingness to pay additional annual taxes to reduce gun injuries. Cook and Ludwig (2002)^20^ next employed this approach to assess the value of safety in a special case estimating costs of gun violence against children.

Finally, several included studies employed a cohort design^21–24^ capturing the economic value lost or changes in costs from firearm injuries among a specific group of individuals. Each study examined a variety of costs attributable to firearm injuries, including productivity costs, family spillover effects, and time to return to employment.

Because studies were observational, they were subject to many sources of potential bias (Appendix Table 3, available online). Only 2 studies included a comparison group, only 1 study blinded investigators to outcomes, and many studies did not identify confounders or adjust for them. However, most studies had good measures of exposure and outcomes and did not have conflicts of interest. Blank cells in Appendix Table 4 (available online) show areas of societal costs that often are missing from these analyses.

A wide range of costs were found in the studies (Appendix Table 4, available online). Although the study focused on nonmedical costs of firearm injuries, 11 studies reported medical costs in addition to other societal costs. Medical costs are briefly summarized in this article for context and comparison.

Costs were reported in terms of aggregate national costs and per-injury costs. Aggregate annual national costs varied from 794 million^19^ to 4.7 billion.^18,12^ Costs per injury varied substantially on the basis of the type of injury, from $1,966 for injuries treated in the emergency department only^18^ to $147,903 for hospitalization and rehabilitation costs for nonfatal injuries.^10^

Overall, these 11 studies generally had medical costs of a similar magnitude to what was found in the systematic review of medical costs of firearm injuries. These studies also had highly variable costs, often varying by the type of injury or injury severity. More details on the medical costs in these articles can be found in the Appendix (available online).

Firearm injuries result in significant utilization of law enforcement, legal, and other criminal justice system resources. Estimated criminal justice costs can be found in Appendix Table 4 (available online).

Incarceration costs of gun violence include pretrial jail detention and subsequent imprisonment. The National Institute for Criminal Justice Reform calculated that detention and incarceration costs are $573,714 per homicide suspect and $219,448 per nonfatal injury shooting suspect.^10^ Follman and colleagues^12^ calculate a national aggregate cost of $7.2 billion per year for long-term prison incarceration for people injuring others with firearms.

Other studies calculate the overall police and criminal justice costs. Children’s Safety Network calculated an aggregate police and criminal justice cost to be 7.04 billion per year,^18^ and Everytown for Gun Safety’s report calculated an aggregate of 13.7 billion in police and criminal justice costs.^9^

The authors acknowledge but do not quantify the economic costs associated with prevention of crime. Reductions in firearm injury risks could potentially reduce the need for police protection and other prevention-associated costs (e.g., metal detectors in public buildings, schools, and athletic events). However, increased prevention efforts could have variable effects on eventual firearm costs depending on what prevention tools are used and how effective they are. Shootings often exhibit low clearance rates in many cities. Although this may reduce immediate criminal-justice-system costs, low clearance rates may also increase future costs of gun violence by undermining deterrence, increasing the probability of retaliatory violence or repeated violence by original perpetrators. Appendix (available online) provides more criminal justice details.

Fourteen papers investigated work/productivity loss due to firearm-related injuries, and 13 of them quantified these losses in dollars. In 2000, Corso et al.^13^ (2006) reported that both fatal and nonfatal firearm injuries resulted in $62.6 billion in lost productivity, primarily affecting individuals aged 15−64 years, whereas Corso and colleagues^14^ (2007) broke out values into $29.5 billion for interpersonal violence and $29.0 billion for selfinflicted cases of firearm injury and death. Children’s Safety Network estimated that the total work loss was $75 billion, with $2.23 million per fatal case; $1.05 million per case requiring hospital admission; and $4,506 per case involving isolated emergency department care without hospital admission.^18^ Fowler et al.^7^ (2015) estimated $59 billion for fatal injuries and $3.7 billion for nonfatal injuries between 2010 and 2012. Another study found that victims’ wage losses were estimated to be $68 billion.^12^ An analysis of lifetime work loss from gunshot wounds to the head found costs of $58 million for hospitalized patients and $268 million for deaths.^24^ Everytown (2022) estimated annual productivity losses from all firearm injuries and deaths at $67 billion.^9^ National Institute for Criminal Justice Reform reported productivity losses of $643 thousand per fatal homicide and $242 thousand per nonfatal case in 17 U.S. municipalities.^10^

Property values could also be adversely impacted by firearm injuries. A study by the Urban Institute (2017) examined the relationship between firearm violence and local economic factors, including housing values.^17^ Using panel regression with sociodemographic controls, they found that each firearm homicide in census tracts in Minneapolis, MN and Oakland, CA was associated with about a $30,000 decrease in average home values. However, these results should be interpreted with much caution because the researcher did not find statistically significant impacts on home prices in the other 3 cities that they examined.

Firearm injuries and deaths can lead to loss of quality of life and length of life, which both have value. The most common approach to valuing the enjoyment of quality of life and length of life lost from firearm injuries is to assign a dollar value to the quality of life and length of life lost from deaths, often called the value of statistical life approach. It has strong theoretical underpinnings and can be an important way to fully capture the benefits of public policies,^25^ with the U.S. federal government using this approach for decades.^26^

Studies quantified the intangible value of life-years lost to gun violence integrated societal willingness to pay for fatality risk reductions, taking into account values of life preservation and risk aversion, in addition to estimated earnings.^27^ Bonne and colleagues^21^ (2020) applied this approach, finding that the community faces a loss of approximately 52 life-years per death, translating into $6.8 million per death. The WISQARS system estimates an intangible cost of $12.1 million per death or $593 billion in total national aggregate costs of firearm fatalities.^6^ Everytown for Gun Safety estimated intangible national costs of both fatal and nonfatal firearm injuries of 607 billion.^9^ Follman et al.^12^ estimated that both fatal and nonfatal firearm injuries led to 234 billion in intangible national costs. Children’s Safety Network estimated intangible costs of fatal firearm fatalities to be $141 billion ($4.5 million per death).^18^ In addition, Peterson and colleagues^8^ (2023) estimated intangible costs of $14.1 to $17.7 million per firearm fatality.^8^

In calculating nonfatal quality-of-life costs, costs were fine tuned on the basis of age, gender, and survival rates, and averages were adjusted through population weighting to ensure accurate estimations,^9^ or they were based on jury awards.^12^ Children’s Safety Network^18^ estimated intangible costs of nonfatal firearm injuries to be between $152 and $410 thousand per injury or a $21 billion national aggregate.

The study by Lemaire^16^ (2005) is an interesting additional analysis, calculating that firearm deaths reduced life expectancy for the average American by 104 days, adding $8.7 billion in total insurance-related costs to the U.S.

Several studies took the approach of calculating overall total costs, not broken out by type. One approach has been to calculate the GDP loss from firearm-related fatalities. Another approach surveyed the general U.S. population about their willingness to pay to reduce firearm injuries.

The analysis by Peters et al.^11^ estimates the overall macroeconomic consequences of firearm-related fatalities in the 36 high- and middle-income countries making up the Organization for Economic Cooperation and Development and find that the U.S. would lose $25 billion in annual GDP. This was more than all the other 35 high-income Organization for Economic Cooperation and Development countries combined.^11^

Cook and Ludwig (2002)^19^ used a novel willingness-to-pay approach to valuing reductions in firearm injuries, surveying over 1,200 American adults about their willingness to pay taxes to reduce gun violence. They found that the willingness to pay to reduce gun violence by 30% was about $45 billion in total or $1.7 million per injury avoided.^19,20^

**Figure 2.**
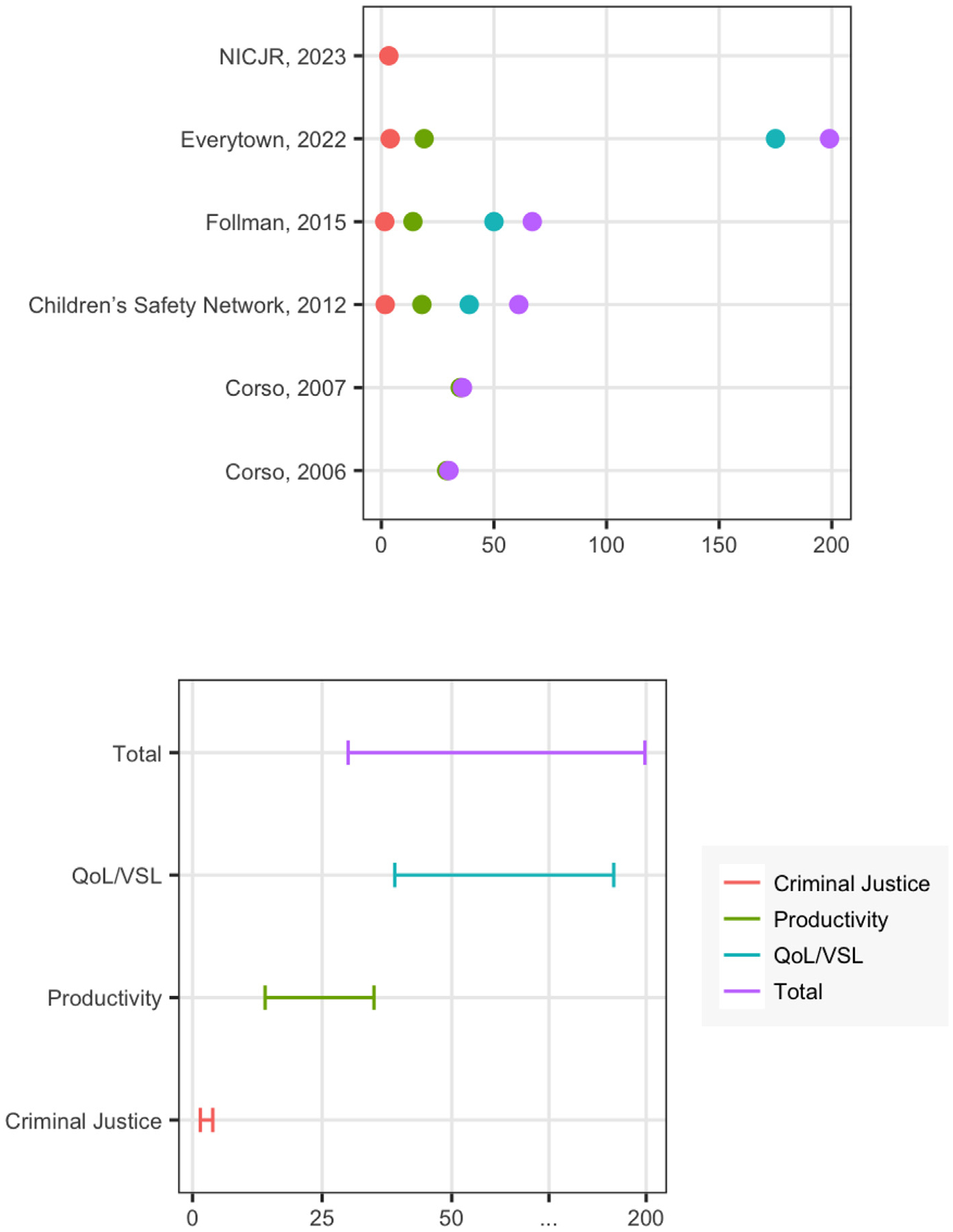
Ratios of nonmedical costs to medical costs. (A) Ratios of nonmedical costs to medical costs. (B) Range of ratios of nonmedical costs to medical costs observed in studies in review.

Because the literature on the medical cost of firearm injury is more robust than that of nonmedical costs, nonmedical costs were compared with medical costs. Studies calculated costs in a variety of settings (e.g., specific cities versus national), time periods, and using different metrics (e.g., per person or national totals), which makes direct comparisons challenging. However, within studies, they typically have similar approaches and populations. Studies that examined both medical and other societal costs found that criminal justice costs were 1.5–3.9 times the value of the medical costs, productivity costs were 14–35 times the value of medical costs, and quality-of-life losses were 39–199 times the magnitude of medical costs (Table 1 and Figure 2).

Table 1 and Figure 2 compare nonmedical costs, including productivity costs, intangible costs, and criminal justice costs, with medical costs. Studies evaluating both fatal and nonfatal injuries are included. Productivity costs are 14–35 times higher than medical costs, quality-of-life or value of statistical life costs are 39–175 times higher than medical costs, criminal justice costs are 1.5–3.9 times higher than medical costs, and the total costs of firearm injuries are 30–199 times higher than medical costs alone.

Of the studies that examined both fatal and nonfatal injuries, costs of fatal firearm injuries were 1.1–17.5 times more costly than those of nonfatal injuries (Table 2). The ratio ranges from 7.4 to 17.5 in national population studies and from 1.1 to 1.9 in per-person studies. Ratios were influenced by the types of costs included, and the study that had the lowest ratio of 1.1 focused on a very specific type of injury: gunshot wounds to the head.

## DISCUSSION

Nineteen studies analyzed nonmedical costs of firearm injuries. Studies generally used modeling approaches (13 studies) to calculate costs, but 4 used a cohort approach, and 2 used a willingness-to-pay approach. Many studies cited a similar source, the work of Ted Miller.

The studies looked at costs of firearms from a variety of perspectives, from aggregate national, state, or city results to individual results. The literature on nonmedical costs of firearm injury evaluated wide ranges of costs —productivity, intangible (quality of life), criminal justice, and other costs—leading to wide ranges of results. Examining results over 2 decades also shows great variation as population and inflation increase. Productivity losses were the most common nonmedical costs studied. Despite having wide ranges of results, studies consistently showed that nonmedical costs were in orders of magnitude larger than medical costs, with total costs potentially in the millions of dollars per injury. In studies evaluating both medical and nonmedical costs, criminal justice costs were 1.5–3.9 times larger, productivity costs were 14–25 times larger, and intangible costs were 29–175 times larger than medical costs. Total costs of firearm injuries are likely in the range of hundreds of thousands to many millions of dollars per person (if they include lost productivity and the value of quality of life lost), and fatal injuries are more costly than nonfatal injuries.

### Limitations

The study of societal nonmedical costs of firearm injury is a relatively small body of literature, especially given its importance. Substantial knowledge gaps remain about nonmedical societal costs of firearm injuries. Limited research funding and lower priority on firearm-specific injury prevention research could be the causes of this gap.^2^ Evaluating costs of firearm injuries is difficult, and existing methodologies are imperfect. It is difficult to isolate costs related to firearm violence from general violence costs, particularly for firearm-specific criminal justice costs. The field would benefit from more recent, more granular, and more nationally representative studies capturing crime-specific policing costs in different contexts. Because of the nature of firearm injuries, many studies do not have comparison groups. However, it may be reasonable to assume that cost would be close to zero if there were no firearm injury. Finally, many studies are descriptive and do not adjust for known or potential confounders. Although there is substantial risk of bias, some studies have attempted to overcome this using methods such as propensity-score matching and difference in differences.^17^

The authors of these studies had some suggestions. The 2019 report on the Economic Cost of Injury in the U.S. further elaborates on the complexity of measuring nondirect costs, such as the value of statistical life and quality of losses, which are challenging to quantify through regular financial transactions.^28^ These costs are often invisible to stakeholders who typically focus on direct costs such as medical care. Moreover, this report called for research into the variations in the value of statistical life based on age and other demographic factors. Fortunately, there is substantial general economic literature on the value of statistical life that could potentially be helpful. Vella and colleagues^29^ (2020) focused on the long-term economic impacts on individuals, such as a significant increase in unemployment rates among firearm injury survivors and mental health burdens that accompany physical recovery. Other studies such as those by Rossin-Slater et al.^30^ (2020), which show increased antidepressant use, and Beland and Kim^31^ (2016), which show decreased student performance, also examine psychological dimensions of firearm injury. However, these were not quantified in economic terms. It suggests a critical area for future research on quantifying the economic impacts of the psychological dimensions of firearm injuries. Together, these articles call for broader considerations that include insurance impacts, quality of life, and the long-term economic and psychological effects on survivors.

There are several additional ways forward. Better data collection systems using more complex survey designs could yield more representative samples, or perhaps using surveillance sites across the U.S. could inform cost estimates. These systems could use something parallel to the KABCO scale applied for motor vehicle crash injuries to better characterize costs associated with different injury severity levels. More importantly, because the value of length and quality of life lost from firearm injuries is so substantial, it is important to accurately estimate these values. Although the review identified only 2 studies using willingness-to-pay methods, a new study by Cook and colleagues^32^ (2025) estimates that U.S. households are willing to pay an average of 744 dollars per year for a 20% reduction in gun violence, corresponding to a national total of 97.6 billion dollars, demonstrating that willingness-to-pay approaches can capture broad societal impacts that traditional cost of injury methods do not measure. The study also shows a substantial increase in public demand for reducing gun violence compared with their previous estimate of 45 billion dollars for a (larger) 30% reduction in interpersonal firearm violence. Incorporating willingness-to-pay approaches would provide a more complete valuation of the societal burden of firearm violence and the benefits of prevention policies. Continued research is needed to refine and expand these methods. Recent work by Miller et al.^33^ has been incorporating these methods. In addition, many other areas of firearm costs were left unquantified, such as spillover effects on families and friends and costs of prevention or impact on broader societal behavior changes.

Knowing the value of injuries prevented can be useful to advocates. Advocates can calculate cost–benefit ratios by comparing the costs of firearm violence prevention programs (e.g., from Schnippel and colleagues^34^ [2003] and O’Toole et al.^35^ [2025]) with benefits of programs, which can be calculated by multiplying the number of firearm injuries prevented by the value per injury prevented (from the studies cited in this article or from tools such as the Everytown gun violence calculator).^36^

## CONCLUSIONS

Nonmedical costs of firearm injury are understudied, but productivity costs may be in orders of magnitude larger than medical costs, and intangible quality-of-life costs are likely even greater, leading to overall costs of firearm injury from hundreds of thousands to several millions of dollars per firearm injury. Fatal injuries have higher societal costs than nonfatal injuries when considering those intangible costs. More research is needed to further understand how firearm injury contributes to nonmedical economic costs as well as how prevention programs can mitigate these societal costs.

## Supplementary Material

1

Supplemental materials associated with this article can be found in the online version at https://doi.org/10.1016/j.amepre.2026.108276.

## Figures and Tables

**Table 1.** Within-Study Comparisons of Nonmedical Costs With Medical Costs

		Costs (dollars)					Ratios			
Study	Population	Medical	Criminal justice	Productivity	QOL/VSL	Total	Criminal justice to medical	Productivity to medical	QoL/VSL to medical	Total to medical
Corso et al.,^13^ 2006	National	2.1 B		62.6 B		65 B		29		30
Corso et al.,^14^ 2007	National	2.0 B		59 B		61 B		35		36
Children’s Safety Network,^18^ n.d.	National	4.8 B	7.1 B	75 B	163 B	251 B	1.5	16	35	53M
Follman et al.,^12^ 2015	National	4.7 B	7.2 B	68 B	234 B	317 B	1.5	14	50	67
Everytown,^9^ 2022	National	3.5 B	14 B	66.8 B	607 B	691 B	3.9	19	175	199
National Institute for Criminal Justice Reform,^10^ n.d.	Per person	108,810	356,951			500,396	3.3			

*Note*: Columns may not add up to totals because the totals may include other cost categories.

All costs are inflated to 2025 U.S. dollars using the GDP deflator.

B, billion; GDP, gross domestic product; M, million; QOL, quality of life; VSL, value of statistical life.

**Table 2.** Within-Study Comparisons of Total Costs Due to Fatal and Nonfatal Injuries

Study	Costs included	Population	Total costs, fatal (dollars)	Total costs, nonfatal (dollars)	Ratio
Corso et al.,^14^ 2007	Medical, productivity	National	56.4 B	3.9 B	14.4
Schoen et al.,^24^ 2023	Medical, productivity	Per person	902,535	819,844	1.1
National Institute for Criminal Justice Reform^10^	Medical, criminal justice	Per person	744,886	402,227	1.9
Finkelstein et al.,^15^ 2006	Productivity	National	59.3 B	3.4 B	17.5
Children’s Safety Network^18^	Medical, productivity, criminal justice, quality of life, employer	National	220.8 B	30 B	7.4
Fowler et al.,^7^ 2015	Medical, productivity	National	61 B	6.0 B	10.2

*Note*: All costs are inflated to 2025 U.S. dollars using the GDP deflator.

B, billion; GDP, gross domestic product.
