# Supplementary material for: Systematic Review of Nonmedical Costs of Firearm Injury
Source: Am J Prev Med. Author manuscript; Available in PMC 2026 Jul 28. (PMC13411774; doi:10.1016/j.amepre.2026.108276)
Supplement: 1 [file NIHMS2189767-supplement-1.pdf]

American Journal of Preventive Medicine

Systematic Review of Non-Medical Costs of Firearm Injury

Appendix

David W. Hutton[1], Taylor W. Lefler[2], Danwei Yang[3], Shiying Mai[4], Michael Holtz[5], Hanwen Zhang[6], Honey S. Modi[7], William B. Hillegass[2], Marc Zimmerman[5,8], Patrick M. Carter[5,8,9]

[1] Department of Health Management and Policy, University of Michigan

[2] University of Mississippi Medical Center

[3] Department of Public Health Sciences, University of Chicago

[4] Division of Social and Administrative Sciences, School of Pharmacy, University of Wisconsin- Madison

[5] Institute for Firearm Injury Prevention, University of Michigan

[6] College of Pharmacy, University of Texas at Austin

[7] Stryker

[8] Department of Health Behavior & Health Equity, University of Michigan

[9] Department of Emergency Medicine, University of Michigan

Appendix Table 1. Summary of Cost Types		
**Cost type**	**Quantified in Dollars**	**Discussed, but not quantified in dollars**
**Medical**	Corso, 2006[13]; Corso, 2007[14]; Children's Safety Network, 2012[18]; Fowler, 2015[7]; Follman, 2015[19]; Bonne, 2020[22];Peterson, 2021[29]; Everytown, 2022[9]; Song, 2022[24]; Peterson, 2023[8]; NICJR, 2023[10];WISQARS, 2022[6]	
**Productivity**	Cook and Ludwig, 2000(MI)[20]; Corso, 2006[13]; Finkelstein, 2006[15]; Corso, 2007[14]; Children's Safety Network, 2012[18]; Follman, 2015[19]; Fowler, 2015[7];Peterson, 2021[29]; Everytown, 2022[9]; Schoen, 2023[25]; Peterson, 2023[8]	Irvin-Erickson, 2017[17]
Family spillover		Fowler, 2015[7]
Employer	Children's Safety Network, 2012[18]; Everytown, 2022[9]	
Tax revenue	NICJR, 2023[10]	Fowler, 2015[7]
Nonmarket		Cook and Ludwig, 2000(MI)[20]; Corso, 2006[13]; Corso, 2007[14]; Follman, 2015[19]
**Criminal Justice**	Children's Safety Network, 2012[18]; Everytown, 2022[9]; NICJR, 2023[10]	
Crime scene response	NICJR, 2023[10]	
Legal	Fowler, 2015[7]	
Incarceration	Follman, 2015[19]; Everytown, 2022[9]	
Victim support	NICJR, 2023[10]	
**Prevention**		
Government prevention	Cook and Ludwig, 2000(MI)[20]; Cook and Ludwig, 2002[21]	
Private prevention	Cook and Ludwig, 2000(MI)[20]	Cook and Ludwig, 2000(MI)[20]
**Value of Statistical Life (quality of life)**	Cook and Ludwig, 2000(WTP)[20]; Children's Safety Network, 2012[18]; Follman, 2015[19];Bonne, 2020[22];Peterson, 2021[29]; Everytown, 2022[9]; WISQARS, 2022[6] McCollister 2010[38]	Fowler, 2015[7]
**Mental Health**	Children's Safety Network, 2012[18]; Pulcini, 2021[23]	Song, 2022[24]
**Other**		
GDP	Peters, 2020[11]	

Insurance claims	Lemaire, 2005[16];Children's Safety Network, 2012[18]	
Credit score		Irvin-Erickson, 2017[17]
Homeownership		Irvin-Erickson, 2017[17]
Home value		Irvin-Erickson, 2017[17]; Fowler, 2015[7]
Emergency transport	Children's Safety Network, 2012[18]	

*Criminal justice system includes police investigation, attorney, defense attorney, court personnel and judges

Appendix Table 2. Summary of Costing Methods

Methods	Studies	Number of studies
Modeling	Lemaire, 2005[16]; Corso, 2006[13]; Corso, 2007[14]; Finkelstein, 2006[15]; Children's Safety Network, 2012[18]; Follman, 2015[12]; Fowler, 2015[7]; Irvin-Erickson, 2017[17]; Peters, 2020[11]; Everytown, 2022[9]; NICJR, 2023[10]; Peterson, 2023[8]; WISQARS, 2022[6]	14
Willingness to pay	Cook and Ludwig 2000[20]; Cook and Ludwig 2002[21]	2
Cohort	Bonne, 2020[22]; Pulcini, 2021[23]; Song, 2022[24]; Schoen, 2023[25]	4

Appendix Table 3: Characteristics Associated with Risk of Bias in Studies

Study (author, year)	Study included a comparison group?	All study groups derived from similar source/reference populations?	Measure of exposure is valid?	Measure of outcome is valid?	Investigators blinded to endpoint assessment?	Potential confounders identified (e.g., comorbidities, multicomponent interventions, etc.)?	Statistical adjustment for potential confounders done?	Funding source(s) disclosed and no obvious conflict of interest?
Corso, 2006[13]	No	No	Yes	Yes	Unsure	No	No	Yes
Corso, 2007[14]	No	Yes	Yes	Yes	No	No	No	No
Follman, 2015[12]	No	Unsure	Yes	Yes	No	No	No	Unsure

**Everytown, 2022**[9]	**No**	**No**	**Yes**	**Yes**	**No**	**No**	**No**	**Yes**
**WISQARS, 2022**[6]	**No**	**Yes**	**Yes**	**Yes**	**No**	**No**	**No**	**Yes**
**Schoen, 2023**[25]	**No**	**Yes**	**Yes**	**Yes**	**No**	**No**	**No**	**No**
**Bonne, 2020**[22]	**No**	**Yes**	**Yes**	**Yes**	**No**	**No**	**Unsure**	**Yes**
**NICJR, 2023**[10]	**No**	**Yes**	**Yes**	**Yes**	**No**	**No**	**No**	**Yes**
**Song, 2022**[24]	**Yes**	**Yes**	**Yes**	**Yes**	**Yes**	**Yes**	**Yes**	**Yes**

**Cook and Ludwig, 2002**[21]	**No**	**Unsure**	**No**	**No**	**No**	**No**	**No**	**Yes**
**Cook and Ludwig 2000**[20]	**No**	**Unsure**	**Yes**	**Yes**	**Unsure**	**Yes**	**Yes**	**Yes**
**Lemaire, 2005**[16]	**No**	**No**	**Yes**	**Yes**	**Unsure**	**Yes**	**No**	**Yes**
**Finkelstein, 2006**[15]	**No**	**No**	**Yes**	**Yes**	**No**	**No**	**No**	**No**
**Children's Safety**	**No**	**Unsure**	**Yes**	**Yes**	**No**	**No**	**No**	**Yes**

**Network, 2012**[18]								
**Fowler, 2015**[7]	**No**	**Yes**	**Yes**	**Yes**	**No**	**Yes**	**Yes**	**Yes**
**Peters, 2020**[11]	**No**	**No**	**Yes**	**Yes**	**Unsure**	**Yes**	**No**	**Yes**
**Peterson, 2023**[8]	**No**	**Yes**	**Yes**	**Yes**	**Unsure**	**No**	**No**	**Yes**
**Pulcini, 2021**[23]	**No**	**Yes**	**Yes**	**Yes**	**No**	**Unsure**	**No**	**Yes**
**Irvin- Erickson, 2017**[17]	**Yes**	**Unsure**	**Yes**	**Yes**	**Unsure**	**No**	**Unsure**	**Yes**

Although these are standard characteristics of bias associated with observational studies, some characteristics like comparisons and blinding may be more relevant to studying interventions that may be more easily blinded or have comparisons and may be much more difficult for evaluation of firearm injury prevention. Clearly, people cannot be randomized to firearm injuries for researchers to have comparisons with extremely similar confounders. However, although finding comparison groups or blinding may be difficult, a lack of comparison group/counterfactual group and blinding could potentially lead to bias. And, although difficult, it may be possible to incorporate blinding and/or find reasonably appropriate comparison groups.

Appendix Table 4: Summary of Firearm Injury Costs in Studies

**Study (author, year)**	**Years**	**Population**	**Violence Types**	**Medical Costs**	**Productivity Costs**	**Employer Costs**	**Intangible (Quality-of-Life or Value of Statistical Life) Costs**	**Prison/Police/Criminal Justice costs**	**Other Costs**	**Total Costs**
Corso, 2006[13]	2000	national incidence in 2000	fatal & non-fatal	2.1 B†	62.6 B†					65 B†
Corso, 2007[14]	2000	national incidence in 2000	fatal: interpersonal	77 M†	27.37 B†					27.44 B†
			fatal: self-inflicted	62 M†	28.8 B†					28.95 B†
			fatal and non-fatal: interpersonal	1,460 M†	29.50 B†					31 B†

			fatal and non-fatal: self-inflicted	221 M†	29.04 B†					29.27 B†
Follman, 2015[12]	2012	national incidence in 2012	fatal and non-fatal	4.7 B†	68 B†		234 B†	7.2 B†	3.3 B†	317 B†
Everytown, 2022[9]	2019	national incidence in 2019	fatal and non-fatal	3.5 B†	66.8 B†	0.62 B†	607.1 B†	14 B†		691 B†
WISQARS, 2022[6]	2022	Nationwide	fatal	333 M†			593 B†			593 B†
				6,908*						12.1 M*
Schoen, 2023[25]	2015	patients with gunshot wounds to the head injuries from October 2013 to October 2015	nonfatal - severely impaired (71 patients)		819,844*					819,844*
			fatal (297 deaths)		902,535*					902,535*
Bonne, 2020[22]	2016	128 recidivists died at the time of their second gunshot wound incident from 2000 to 2017		83,726*	1.72 M*		6.8 M*			8.7 M*
NICJR, 2023[10]	2021	17 municipalities in US	fatal-homicide	11,450*				643,380*	lost revenue: 90,054*	744,886*

			nonfatal	147,903*				241,941*	lost revenue: 12,538*	402,227*
Song, 2022[24]	2019	6498 firearm injuries survivors from 2008 to 2018	nonfatal - unintentional	1-year post-injury changes: 2,204* (1,833-2,577))						2,204* (1,833-2,577)
			nonfatal - intentional	1-year post-injury changes: 5,178* (4,273-6,085)						5,178* (4,273-6,085)
		12,489 family members of survivors from 2008 to 2018	nonfatal - unintentional	1-year post-injury changes: 9* (-37 - 55)						9* (-37 - 55)
			nonfatal - intentional	1-year post-injury changes: 42* (-46 - 129)						42* (-46 - 129)
Cook and Ludwig, 2002[21]	1998	total number of households in the US							WTP $45 B to reduce assault-related gunshot injuries by 30%.	
Cook and Ludwig 2000A (Medical Chapter)[2]	1998	Nationwide	total fatal and nonfatal	794 M - 2.2 B†						

0										
Cook and Ludwig, 2000B (WTP Chapter)[20]	1998	nationally surveyed people's WTP to reduce gun violence by 30% in 1998	avoid assaults						43.7 B†	129 B† 1.66 M*
			avoid self-inflicted						37 B†	
			avoid unintentional						8.1 B†	
Cook and Ludwig, 2000C (Productivity Chapter)[20]	1998	gunshot wounds in 1997	homicide		882,093*					870,405*
			suicide		866,445*					
			unintentional		780,724*					
Lemaire, 2005[16]	2000	firearm death	homicides				45.9 days*		1.44§	
			suicides				52.3 days*		1.41§	
Finkelstein, 2006[15]	2000	national incidence in 2000	fatal		59.251 B†					59.251 B†
			nonfatal		3.433 B†					3.433 B†
Children's Safety Network, 2012[18]	2010	national incidence in 2010	fatal	1.832 B† 57,858*	70.82 B† 2.236 M*	409.672 M† 12,930*	141.14 B† 4.456 M*	6.454 B† 572,331*	Insurance claim processing: 107.71 M†, 3,401*	220.764 B† 7.339 M*
			hospital admitted	2.806 B† 72,761*	4.036 B† 104,650*	122.43 M† 3,175*	16.457 B† 427,078*	295.368 M† 10,930*	Insurance claim processing: 186.371 M† 4833*	23.917 B† 623,426*

			emergency department only	68,696 M† 1,966*	157.421 M† 4506*	17.815 M† 510*	5.543 B† 158,629*	292.62 M† 10,350*	Insurance claim processing : 28.787 M† 824*	6.110 B† 177,148*
Fowler, 2015[7]	2010-2012	annual national incidence between 2010 and 2012	fatal	596 M† 18,303*	58.9 B† 1.8 M*					59.659 B† 1.8 M*
			nonfatal	1,050 M† 15,639*	3.7 B† 55,487*					5.892 B† 71,126*
Peters, 2020[11]	2030	modeling GDP loss in the US in 2030	fatal						GDP: $25.4 B†	$25.4 B†
Peterson, 2023[8]	2020	2020 2020	Firearm - Suicide	4,968*			12 M*‡			12 M*
			Firearm - Homicide	11,030*			14.3 M*‡			14.3 M*
			Firearm - Legal Intervention	8,520*			13.7 M*‡			13.7 M*
			Firearm - Unintentional	17,283*			15 M*‡			15 M*
			Firearm - Undetermine d	12,658*			14.5 M*‡			14.5 M*
			Firearm- All	7,827*			13.1 M*‡			13.1 M*
Pulcini, 2021[23]	NR	3,296 children with firearm injuries from 2010	Non-fatal						mental health expenditur e increase after 1 year of	$337*

		to 2016							injury: $337*	
Irvin-Erickson, 2017[17]	2017	6 US Cities from 2009-2015	fatal and nonfatal						various neighborhood business and home value, homeownership, and credit score outcomes, but these varied by city and were often not statistically significant. ‖	

* per-person

† total

‡ all non-medical costs, but a majority are monetized quality-of-life losses

§ The insurance net single premium reduction per $1,000 of coverage that could be reduced if firearm injuries are avoided in a 20-year, fully discrete whole life insurance policy for a 25-year-old at the time of issue.

‖ Two statistically-significant results were for home values in two cities: a $28,338 drop in home values in census tracts per firearm homicide in Minneapolis, Minnesota, and a $31,770 drop in home prices in census tracts in Oakland, California. Extreme care should be taken in generalizing these results since the results for the three other cities studied were not statistically significant.

All costs inflated to 2025 US dollars using the GDP deflator

Gun Violence Nonmedical Economic Costs

NR: not reported, WTP: willingness-to-pay

Additional Details on Medical Costs

The 11 studies identified in the review often reported costs in terms of aggregate national costs and not per-injury costs. All costs are reported in 2025 dollars inflated using the GDP deflator.

A study by Corso et al in 2006 on costs of injuries found that the total lifetime medical costs of firearm injuries in 2000 was $2.18 billion overall or $16,638 per injury.[13] In 2007, Corso and others produced another study on a subset of those injuries, this time focusing on interpersonal and self-directed violence (excluding unintentional injuries) in 2000.[14] They found total medical costs to be $1.463 billion ($27,213 per injury event) for interpersonal violence and $221 million for self-inflicted violence ($11,892 per injury event) for a total of $1.683 billion or $23,284 per injury.

A study by Cook and Ludwig (2002) estimates the net medical costs of gun violence to be between 794 million and 22 billion per year.[20] This accounts for the 3.5 billion in medical costs directly associated with the injury, but then subtracts reductions in medical costs from other injuries and lifetime healthcare costs averted because of the death caused from the firearm.

In a study of a hospital in New Jersey from 2000-2017, Bonne et al. (2020) found mean hospital costs of $83,726.[22]

The WISQARS system and the Pacific Institute for Research and Evaluation (PIRE) model were frequently used (the PIRE model is integrated into WISQARS). The Children's Safety Network in 2012 used WISQARS data for incidence and the PIRE model for injury costs to estimate total medical costs of fatal firearm injury (physical, mental, and emergency transport) to be $1.9 billion, medical costs of nonfatal firearm injuries to be $2.9 billion in 2010.[18]

Also, using data from WISQARS, Fowler et al. (2015) does not specifically present medical costs, but notes that of the total costs of fatal firearm injury, 99% of the costs result from lost productivity costs (compared to 1% medical), and that 61% -79% of costs of nonfatal firearm injuries are lost productivity costs (compared to 21% -29% medical).[7]

Follman et al (2015), also using the PIRE Model to calculate the annual cost of gun violence in America, estimated medical costs to be $4.7 billion in 2015.[19]

In a study using the WISQARS cost of injury system, Peterson et al. (2023) found firearm-related medical costs in 2020 to be $9,223 per person and (non-medical costs to be 15.4 Million).[8]

The Everytown Support Fund for Gun Safety used WISQARS and PIRE to calculate that in 2019, there were $3.5 billion in medical costs related to firearm injury.[9]

The CDC WISQARS database can be queried directly and they estimate that in 2022, each *fatal* firearm incident caused an average of $6,918 in medical costs for a national total of $333 million dollars.[6] WISQARS currently recommends against using their data for nonfatal firearm injury prevalence because of limitations in their hospital sampling methodology.

The National Institute for Criminal Justice Reform (NICJR) created a study in 2023 that calculated the costs of shootings in 17 U.S. municipalities.[10] They calculated the costs of hospitalization and rehabilitation to be on average $11,452 per homicide and $147,903 per non-fatal shooting injury.

A study by Song et al. in 2022 used a different approach.[24] They matched firearm injury survivors and their family members with control insurance plan members and found that survivors had $37,161 in higher costs in the year following their firearm injury. Family members had $194 in higher spending, but this was not statistically significantly different from zero (p=0.37).

Some studies broke out medical insurance costs separately. In Cook and Ludwig (2000), the authors utilize hospital discharge data from two states to estimate the medical costs, and specifically, claims administration costs, of treating fatal and nonfatal firearm injuries.[20] In Maryland 1994-95, there were 2,394 nonfatal hospitalized firearm injury cases. For those, the claims administration costs ranged from $3,061 to $6,020 across injury intents. Additionally, there were 200 fatal hospitalized firearm injury cases for which the claims costs ranged from $746 to $1,187 across injury intents. In New York 1994, there were 3,334 nonfatal hospitalized gunshot injury cases. For those, the claims administration costs ranged from $3,537 to $11,126 across injury intents. There were also 258 fatal hospitalized firearm injury cases for which the claims costs ranged from $614 to $1,830 across injury intents.

Finally, a few studies broke out mental health costs from other health costs. There is a paucity in research on specific costs related to mental health treatment following firearm injuries. Furthermore, the costs that are reported are likely underestimated as a significant proportion of firearm injury victims and their families either are unable to access or cannot afford to seek counseling. Those services might not even be available in some communities. A report by Follman et. al. (2015) in collaboration with Miller[19] used the rates of people who sought counseling and the related costs from his previous study[39] and applied them

to the current data on fatal and nonfatal firearm injuries. Doing so, he estimated an annual $568 million in direct costs for mental health treatment.

Based on mental health claims associated with nonfatal firearm injuries of 3,926 children 0-18 years old from the Medicaid MarketScan claims database from 2010 to 2016, Pulcini et al. (2021) evaluated three subgroups: low prior healthcare expenditures (previously healthy), high prior outpatient mental health expenditures (>90th percentile=$1,866), and high prior non-mental health expenditures (>90th percentile=$6,730).[23] They reported a significant decrease in the mental health expenditures (standard payment) comparing one year before and one year after the firearm injury for the overall cohort, but children with low prior expenditures had a significant increase in expenditures after a firearm injury. Conversely, a significant decrease in expenditures was observed among those with high prior mental health expenditures and high non-mental health prior expenditures.

The Children's Safety Network estimated mental healthcare costs to be about 11% of the healthcare system costs of firearm injury, but less than 1% of overall societal costs of firearm injury.[18]

Additional Details on Criminal Justice Results

Gun violence incidents, injuries, and deaths result in significant utilization of law enforcement and the criminal justice system resources. More specifically, this systematic review focuses on economic costs attributable to gun violence injuries and deaths. In addition to producing aggregate cost estimates, the objective is to produce unit average cost estimates of law enforcement and criminal justice system costs per gun violence nonfatal injury or death (homicide). If an intervention reduces the number of gun violence injuries and deaths, an estimate of the law enforcement and criminal justice system cost inputs (both labor and capital) which can be decreased or reallocated to another use would likely be an important element in assessing benefit compared to the cost of the intervention. Unavoidably, the systematic review is also intended to gauge the completeness, accuracy, and limitations of the available data and analysis to estimate these quantities.

The systematic review initially identified six publications addressing law enforcement and criminal justice system costs of injuries and deaths related to violence. These six identified publications included only one reporting original primary data and results.[10] The others were based upon five unique primary sources[40–44]. These five primary sub-sources were identified through review of six publications and all their references from the year 2000 to present. These key underlying references were not initially identified in the systematic review due to broader scopes than gun violence injuries and deaths alone. However, these papers are included here in the appendix.

Estimation of law enforcement and criminal justice system costs of gun violence injuries and deaths requires consideration of several methodological issues.

First, most studies use either a “top-down” or “bottom-up” cost accounting approach. The top-down approach starts with total expenditures and then apportions these between labor and supplies/equipment followed by calculating the shares of each of these in producing specific services. Often there is limited data to accurately estimate these shares. Subsequently apportioning these to services responding to particular crime types introduces further uncertainty. Most studies follow the Federal Bureau of Investigation Uniform Crime Reporting system with Part 1 crimes being violent and Part 2 non-violent. The reporting system does not parse Part 1 crimes, including homicide and aggravated assault, as to whether a firearm was involved let alone injury sustained. Bottom-up studies attempt to

identify and quantify the resources required to respond to each specific crime type (general police office hours, detective hours, non-sworn personnel hours, testifying in court time) and then determine per unit cost of those resources (hourly pay, overhead, etc.) These bottom-up methodologies may underestimate the full costs associated with firearm violence because they miss infrastructure costs and surge capacities required to maintain these systems. As with top-down studies, sufficient empirical data tends to be unavailable to guarantee accuracy. But a combination of approaches may help triangulate in on the true costs or at least provide bounds on what the costs might be.

Second, the law enforcement and criminal justice system services are non-market services produced without charged prices per unit produced. Hence, budgets and expenditures for labor, equipment, and supplies must be apportioned. Most studies estimate the variable costs. These are labor and equipment/supplies (patrol cars, communications devices, weapons, crime scene kits, etc.) but not fixed costs. Hence, facilities and other major capital outlays are not included. Hence, the estimates are likely informative in the short-run of several years but not in the long-run of a decade or more. Given that labor is the largest cost component, it is also not the case that less crime results in lower variable costs in the short-run. Hence, reducing crime may paradoxically result in higher average estimated costs per unit crime. Further, approximately 50% of law enforcement labor time is dedicated to tasks unrelated to specific crimes such as traffic control, jail security, and preventive patrolling. This time and expenditure is typically and appropriately excluded in estimating the law enforcement cost per crime.

Third, even similar crime incidents do not uniformly precipitate the same sequence of sequelae such as arrest, trial, conviction, and incarceration. For example, “clearance rates” of crimes charged versus reported are only 54% for homicide in the US in 2020. Clearance rates are considerably lower for other violent crimes. This results in additional variability in law enforcement and criminal justice system cost metrics reported. These range from average cost per offender convicted, per suspect charged, per subject arrested, or per crime reported resulting in high variability of the denominator (divisor) given cost estimates.

Fourth, law enforcement and criminal justice system resource utilization vary several fold by crime type including crime scene analysis, investigation, court time, and prosecutor/defender time as well as incarceration likelihood and duration. Further, the mix of crime types varies considerably from jurisdiction to jurisdiction particularly between urban and rural areas.

Fifth, perhaps given these complexities, studies estimating law enforcement and criminal justice system costs for specific crimes particularly resulting in gun violence injuries and deaths are infrequently performed and reported.

Reports of resource utilization include initial crime scene law enforcement and emergency medical services (ambulance/fire department) response. The typical initial response activities include crime scene control, scene investigation and processing, arrests, and crime scene cleanup. Subsequent law enforcement phases include investigations and testifying. The systematic review distilled essentially two unique sources that estimated these costs.

Hunt et al. published two works [40,41] utilizing a top-down approach to calculate law enforcement costs, judicial costs, and criminal legal costs. Although these are very thorough analyses of monetized potential benefit for criminal justice services of crime, they are not necessarily specific to firearm crimes. Yet, because 60 to 80% of homicides involve a firearm these estimates likely provide the closest available estimates of law enforcement resource costs per gun violence death[45].

In the 2017 study of judicial and legal costs, the authors found costs of $46,541 per murder (10th percentile of $18,038 and 90th percentile of $76,555).[40] In their subsequent 2019 article on law enforcement costs, they found homicide has an average law enforcement costs of $227,895 per homicide with 10th percentile of $170,602 and 90th percentile of $364,905.[41] Aggravated assault as a surrogate for violent nonfatal firearm injuries in 2010 dollars has a mean estimate of $14,774 with 10th percentile $5,031 and 90th percentile of $25,579. Their sophisticated model estimates "state-specific variable costs of Part 1 crimes by crime type for law enforcement services."[41] Direct current expenditures for local and state law enforcement are extracted from the Justice Expenditure and Employment Extracts 2010 from the U.S. Bureau of Justice Statistics.[46] Corrections facilities' data is excluded. All labor (sworn and non-sworn) costs and operating expenses (equipment/supplies purchases) aggregate numbers are utilized. Relative dollar wages are standardized across jurisdictions utilizing the Bureau of Labor Statistics index of public administration wages. Deadweight losses due to taxation are applied to payroll expenditures.[47] The proportion of time spent in crime-response activities by officer role are obtained from the Law Enforcement Management and Administration Statistics (LEMAS)[48] which surveys state, local, and sheriff's departments and seven state-specific additional studies using time diary or observational approaches. US Census data is applied to adjust for the degree of urbanization. Hours of police time spent by FBI UCR Part 1 crime type are then applied to the labor time data. These estimates of police time by crime type

are derived from data collected in Houston, TX and Vermont. Houston data is applied to urban areas and Vermont to rural areas. Hunt et al. discuss in detail limitations of this data and efforts to mitigate the use of information from two sites as a "proxy for multiple locations" in accounting for the likely uncertainty of this limited primary data. The FBI UCR 2010 number of crimes reported to police by jurisdiction are used as the denominators to estimate resource use per reported crime. Finally, a Monte Carlo simulation is performed for each crime type for every to simultaneously probabilistically account for the underlying uncertainty in all incorporated estimates to yield mean, minimum, maximum, 10th percentile, and 90th percentile estimates of law enforcement resource cost per crime type for each state.

Separate from the work by Hunt et al, the National Institute for Criminal Justice Reform (NICJR) used a mixture of bottom-up and top-down approaches to estimate criminal justice costs from 17 cities they felt may be broadly representative of the United States.[10] They estimated that crime scene response, police investigation, crime scene cleanup, and medical examiner costs led to $23,622 cost per homicide and $11,742 per nonfatal assault injury. They estimated court and legal costs to be $27,749 per homicide and $3,518 per nonfatal firearm shooting.

The NICJR methods necessarily vary somewhat from city to city based on available data sources. Most data sources appear to reflect 2020 to 2022 dollars. Specific approaches and calculations for each city are detailed. For most cities the NICJR method uses police and EMS expenditures and salaries from budgets and financial reports. The mix of law enforcement officer type and average hours required for fatal and nonfatal shootings are then estimated. The average cost among competitive crime scene cleanup vendors is recorded. Police department data was used and/or interviews were performed with active and/or retired police personnel to estimate subsequent investigative time by law enforcement officer type multiplied by hourly salary expenses.

The NICJR costs are realized and estimated in several discrete phases.

1. Crime scene response phase costs. Utilizing local police department budgets, payrolls, schedules, and interviews, the estimated police crime scene response resource cost was $2,403 per nonfatal gunshot injury and $3,694 per gun violence homicide. Similarly, emergency medical services (fire and ambulance) costs were tallied and estimated from city budgets, annual reports and vendor contracts. 1st responder costs were estimated at $2,332 per homicide or nonfatal injury shooting. This leads to an estimated unit cost of $4,735 per nonfatal injury shooting for police and 1st responder response and an estimated cost of $6,026 for this response to a fatal shooting.

2. Police investigation phase costs. Similarly, from local police department interviews budgets and payroll schedules, The National Institute for Criminal Justice Reform estimated an average cost of $4,027 per nonfatal injury shooting police investigation and $11,636 per homicide investigation phase occurring after the initial crime scene phase. There is limited to no data on the frequency with which the police investigation phase transpires as a proportion of crime scene responses.

3. Crime scene cleanup phase. Data from crime scene cleanup vendor contracts and interviews yielded an average estimate of $2,980 per homicide and nonfatal injury shooting.

4. Medical examiner/corner costs. Based on data from office of medical examiners websites as well as interviews, the estimated cost of medical examiner services is also $2,980 per homicide.

The NICJR has also provided estimates of the courts and legal system response to gun violence resulting in fatal and non-fatal gunshot injuries. The legal system response to gun violence injuries, and homicides includes multiple resource costs. Hunt et al. noted in 2017 in the American Journal of Criminal Justice, "Remarkably, however, we currently lack measures of how much specific crimes cost taxpayers through the legal system across state jurisdictions. While the costs of providing judicial and legal services for individual crimes are relatively small compared to the overall harm that crimes cause, they are still quite significant. Since 2007, U.S. taxpayers have provided over $50 billion annually for courts, prosecution, and public defense of all case types." (reported in 2010 dollars) [40,49]

As with law enforcement costs, multiple challenges arise in estimating these costs. They are usually non-market government services or outputs provided without charge thus resulting in no available market prices. Mixtures of services, e.g., civil and criminal cases in courts, misdemeanors versus felonies, or traffic regulation versus criminal cases for law enforcement agencies for example, limit the ability to easily apportion budgeted resources and expenditures to specific activities and crimes. Hence, estimating the opportunity costs of not preventing crime to inform optimal societal resource allocation for court and legal costs also remains methodologically challenging. For the gun violence deaths and injuries, the NICJR identified the following legal (court and prosecution/defense) system costs.[10]

1. Court costs. Again, based on the 17 cities evaluated, judicial salaries as well as court staff and personnel data were used along with the weighted caseload attributable to gun violence injuries and gun violence homicides to estimate court costs per gun violence injury and homicide incident. These were estimated at $10,513 per homicide and $576 per nonfatal injury shooting.

2. Prosecution and defense costs. These were similarly assessed based on workload studies as well as district attorney and public defender budgets and payrolls. These legal costs were estimated at $17,236 per homicide and $2,942 per nonfatal injury shooting.

However, the NICJR's estimates tend to have considerable variance from three studies that have estimated judicial and legal costs by FBI crime type. While these three studies were not identified in the systematic review, they were located in review of all the references of identified studies addressing court and legal costs. In fact, these underlying sources provided the primary data for the systematic review identified references except the NICJR analysis.

Several state-based analyses were identified. Using a top-down methodology apportioning overall expenditures to specific crimes, Aos et al. estimated judicial and legal marginal costs per homicide conviction in Washington state at $246,848.[42] Fowles and Nyström (2012) estimated this marginal cost at $91,120 per offender in Utah also using a top-down methodology.[43] The Vermont Center for Justice Research estimated the average law enforcement opportunity cost per homicide case in Vermont at $52,038 using a bottom-up approach which attempts to identify all resource inputs.[44] Unfortunately, these estimates are not directly comparable due to different denominators of per conviction, per offender, and per charged homicide. For aggravated assault as best potential surrogate for non-fatal gunshot injury cases, Aos et al. (2006) estimated $2,937,[42] Fowles and Nyström (2012) $7,995,[43] and VCJR (2014) $3,957 as the judicial and legal costs but again with disparate methodologies and definitions (e.g., marginal, average, vs. variable costs and per conviction, offender, or case charged). These three studies highlight the limited available data and methodological complexities of quantifying and estimating the true judicial and legal economic costs of gun violence.

Incarceration costs. Incarceration costs of gun violence include pretrial detention jail costs as well as subsequent state prison costs. Jail budgets, particularly payroll costs for local facilities as well as state prisons, were used to estimate costs by the National Institute for Criminal Justice Reform. Largely per incarceration day and year costs for jails and prisons were estimated multiplied by average lengths of pre-trial detention and prison sentences served. Average pre-trial jail time was 730 days between shooting and trial. Corrections department general fund budgets and state prisons ADP data to calculate average inmate cost per year then 25 years prison-time for average homicide and 10 years prison-time for non-

fatal injury shooting. Prison costs are estimated at $573,714 per homicide and $219,448 per nonfatal injury shooting. [10]

1. Pretrial detention costs. The weighted proportion for inmates and detainees charged or convicted of gun violence homicide or shootings was applied to these budgets. Pretrial detention costs were estimated at $74,155 per homicide suspect and $70,384 per nonfatal injury shooting suspect.

2. Post-trial incarceration costs. Similarly, state prison average costs were estimated at $499,558 per homicide convict and $149,063 per nonfatal injury perpetrator convict.

The relationship between gun violence homicides and non-fatal shootings and subsequent utilization of court and incarceration resources is not well documented in the published literature. Specifically, “clearance rates” of these cases are not well documented. This refers to gun violence homicides and shootings where the perpetrator cannot be identified and prosecuted. Given this, unit costs can be estimated with the top-down and bottom-up methods but extrapolation to overall impact on societal resource utilization requires assumptions concerning these clearance rates. In 2022 the homicide clearance rate in the United States was approximately 50% with almost half of homicides going unsolved with no perpetrator identified or arrested. Of those arrested for homicide, fewer than half are convicted. The clearance rate for homicide is higher than for nonfatal shootings.

The criminal justice system has additional costs of crime in general but not attributable to specific crime incidents. Some of these are largely attributable to gun violence. For example, the need to protect law enforcement officers with bulletproof vests is estimated to cost approximately $100 million per year.

Appendix Table 5: Studies of criminal justice costs related to homicides, but that are not specific to firearms

**Study (author, year)**	**Years**	**Population**	**Violence Types**	**Productivity Costs**	**Intangible (Quality-of-Life or Value of Statistical Life) Costs**	**Prison/Police/Criminal Justice costs**
Aos, 2006[42]	2006	Washington State	fatal-homicide	2,120,666	3,935,069	307,927
McColister, 2010[38]	2008	Nationwide	fatal-homicide	1,659,539	12,399,580	576,285

Fowles, 2012[43]	2008	Utah	fatal-homicide	1,083,260		142,680
VCJR, 2014[44]	2012-2013	Vermont	fatal-homicide	1,003,470	11,459,020	32,212
Hunt, 2017[40]	2010	Nationwide	fatal-homicide			46,617*
Hunt, 2019[41]	2010	Nationwide	fatal-homicide			227,895 †

All costs are per-person costs.

All costs are inflated to 2025 US dollars using the GDP deflator

* Judicial and Legal

† Law Enforcement

References

1. Centers for Disease Control and Prevention, National Center for Injury Prevention and Control. Mapping Injury, Overdose, and Violence Dashboard (version date: September 5, 2025). Accessed November 18, 2025. https://www.cdc.gov/injury-violence-data/data-vis/index.html

2. Jaffe S. Decisions to be made on US gun violence research funds. The Lancet. 2020;395(10222):403-404. doi:10.1016/S0140-6736(20)30303-2

3. Society for Advancement of Violence and Injury Research. Funding at Risk: Advocating for Injury and Violence Prevention in Congress. October 4, 2025. Accessed November 18, 2025. https://thesavir.org/newsletter/funding-at-risk-advocating-for-injury-and-violence-prevention-in-congress/

4. Maya Brownstein. Gun violence and injury prevention efforts stymied by federal funding cuts | Harvard T.H. Chan School of Public Health. September 17, 2025. Accessed November 18, 2025. https://hsph.harvard.edu/news/gun-violence-and-injury-prevention-efforts-stymied-by-federal-funding-cuts/

5. Miller T, Downing J, Wheeler L, Fischer K. The Medical Costs of Firearm Injuries in the United States: A Systematic Review. J Emerg Med. 2024;66(2):109-132. doi:10.1016/j.jemermed.2023.08.013

6. WISQARS Cost Of Injury. Accessed June 28, 2024. https://wisqars.cdc.gov/cost/

7. Fowler KA, Dahlberg LL, Haileyesus T, Annest JL. Firearm injuries in the United States. Prev Med. 2015;79:5-14. doi:10.1016/j.ypmed.2015.06.002

8. Peterson C, Rice KL, Williams DD, Thomas R. WISQARS Cost of Injury for public health research and practice. Inj Prev. 2023;29(2):150-157. doi:10.1136/ip-2022-044708

9. Everytown Research & Policy. The Economic Cost of Gun Violence. Accessed June 27, 2024. https://everytownresearch.org/report/the-economic-cost-of-gun-violence/

10. National Institute for Criminal Justice Reform. The National Cost of Gun Violence: The Price Tag for Taxpayers. Accessed June 27, 2024. https://search.issuelab.org/resource/the-national-cost-of-gun-violence-the-price-tag-for-taxpayers.html

11. Peters AW, Yorlets RR, Shrime MG, Alkire BC. The Macroeconomic Consequences Of Firearm-Related Fatalities In OECD Countries, 2018–30: A Value-Of-Lost-Output Analysis. Health Aff (Millwood). 2020;39(11):1961-1969. doi:10.1377/hlthaff.2019.01701

12. The True Cost of Gun Violence in America – Mother Jones. Accessed June 27, 2024. https://www.motherjones.com/politics/2015/04/true-cost-of-gun-violence-in-america/

13. Corso P, Finkelstein E, Miller T, Fiebelkorn I, Zaloshnja E. Incidence and lifetime costs of injuries in the United States. Inj Prev. 2006;12(4):212-218. doi:10.1136/ip.2005.010983

14. Corso PS, Mercy JA, Simon TR, Finkelstein EA, Miller TR. Medical Costs and Productivity Losses Due to Interpersonal and Self-Directed Violence in the United States. Am J Prev Med. 2007;32(6):474-482.e2. doi:10.1016/j.amepre.2007.02.010

15. Finkelstein E, Corso PS, Miller TR. The Incidence and Economic Burden of Injuries in the United States. Oxford University Press; 2006.

16. Lemaire J. The Cost of Firearm Deaths in the United States: Reduced Life Expectancies and Increased Insurance Costs. J Risk Insur. 2005;72(3):359-374. doi:10.1111/j.1539-6975.2005.00128.x

17. Irvin-Erickson Y, Lynch M, Gurvis A, Mohr E, Bai B. A Neighborhood-Level Analysis of the Economic Impact of Gun Violence. Policy Commons; 2017. Accessed June 27, 2024.

https://policycommons.net/artifacts/631561/a-neighborhood-level-analysis-of-the-economic-impact-of-gun-violence/1612871/

18. Children's Safety Network. The Cost of Firearm Violence. Accessed June 27, 2024. https://www.childrenssafetynetwork.org/resources/cost-firearm-violence

19. Follman M, Lee J, West J. The True Cost of Gun Violence in America. Mother Jones. Published online April 15, 2015. Accessed December 2, 2024. https://www.motherjones.com/politics/2015/04/true-cost-of-gun-violence-in-america/

20. Cook PJ, Ludwig J. Gun Violence: The Real Costs. Oxford University Press; 2000.

21. Cook PJ, Ludwig J. The Costs of Gun Violence against Children. Future Child. 2002;12(2):87-99. doi:10.2307/1602740

22. Bonne S, Tufariello A, Coles Z, et al. Identifying participants for inclusion in hospital-based violence intervention: An analysis of 18 years of urban firearm recidivism. J Trauma Acute Care Surg. 2020;89(1):68. doi:10.1097/TA.0000000000002680

23. Pulcini CD, Goyal MK, Hall M, et al. Mental Health Utilization and Expenditures for Children Pre–Post Firearm Injury. Am J Prev Med. 2021;61(1):133-135. doi:10.1016/j.amepre.2021.01.024

24. Song Z, Zubizarreta JR, Giuriato M, Paulos E, Koh KA. Changes in Health Care Spending, Use, and Clinical Outcomes After Nonfatal Firearm Injuries Among Survivors and Family Members. Ann Intern Med. 2022;175(6):795-803. doi:10.7326/M21-2812

25. Schoen N, Matichak D, Armstrong V, et al. The Cost of Gunshot Wounds to the Head: An Unevenly Distributed Burden. World Neurosurg. 2023;172:e201-e211. doi:10.1016/j.wneu.2022.12.130

26. Viscusi WK. Pricing Lives: International Guideposts for Safety. Econ Rec. 2018;94(S1):1-10. doi:10.1111/1475-4932.12396

27. Robinson L, Hammitt J, Baxter J. GUIDELINES FOR REGULATORY IMPACT ANALYSIS 2016. Office of the Assistant Secretary for Planning and Evaluation U.S. Department of Health and Human Service; 2017:95. Accessed December 3, 2024. https://aspe.hhs.gov/sites/default/files/migrated_legacy_files//171981/HHS_RIAGuidance.pdf

28. Viscusi WK. How to value a life. J Econ Finance. 2008;32(4):311-323. doi:10.1007/s12197-008-9030-x

29. Peterson C, Miller GF, Barnett SBL, Florence C. Economic Cost of Injury — United States, 2019. MMWR Morb Mortal Wkly Rep. 2021;70(48):1655-1659. doi:10.15585/mmwr.mm7048a1

30. Vella MA, Warshauer A, Tortorello G, et al. Long-term Functional, Psychological, Emotional, and Social Outcomes in Survivors of Firearm Injuries. JAMA Surg. 2020;155(1):51-59. doi:10.1001/jamasurg.2019.4533

31. Rossin-Slater M, Schnell M, Schwandt H, Trejo S, Uniat L. Local exposure to school shootings and youth antidepressant use. Proc Natl Acad Sci. 2020;117(38):23484-23489. doi:10.1073/pnas.2000804117

32. Beland LP, Kim D. The Effect of High School Shootings on Schools and Student Performance. Educ Eval Policy Anal. 2016;38(1):113-126. doi:10.3102/0162373715590683

33. Cook PJ, Jeuland M, Ludwig J. Valuing the benefits of reducing firearm violence in the United States. Proc Natl Acad Sci. 2025;122(4):e2419864122. doi:10.1073/pnas.2419864122

34. Miller GF, Barnett SBL, Florence CS, McDavid Harrison K, Dahlberg LL, Mercy JA. Costs of Fatal and Nonfatal Firearm Injuries in the U.S., 2019 and 2020. Am J Prev Med. 2024;66(2):195-204. doi:10.1016/j.amepre.2023.09.026

35. Schnippel K, Larson B, Jay J, Szkola J, O'Toole MJ. Costs of Summer Youth Employment to Prevent Violence: an Analysis and Implementer's Tool. J Urban Health. 2023;100(4):676-685. doi:10.1007/s11524-023-00753-8

36. O'Toole MJ, Schnippel K, Larson B. Hospital-based violence intervention programs: An analysis of costs and key components. J Trauma Acute Care Surg. 2025;98(4):655. doi:10.1097/TA.0000000000004498

37. Calculate the Economic Cost of Gun Violence. Everytown Research & Policy. Accessed November 19, 2025. https://everytownresearch.org/report/economic-cost-calculator/

38. McCollister KE, French MT, Fang H. The cost of crime to society: New crime-specific estimates for policy and program evaluation. Drug Alcohol Depend. 2010;108(1-2):98-109. doi:10.1016/j.drugalcdep.2009.12.002

39. Cohen MA, Miller TR. The Cost of Mental Health Care for Victims of Crime. J Interpers Violence. 1998;13(1):93-110. doi:10.1177/088626098013001006

40. Hunt P, Anderson J, Saunders J. The Price of Justice: New National and State-Level Estimates of the Judicial and Legal Costs of Crime to Taxpayers. Am J Crim Justice. 2017;42(2):231-254. doi:10.1007/s12103-016-9362-6

41. Hunt PE, Saunders J, Kilmer B. Estimates of Law Enforcement Costs by Crime Type for Benefit-Cost Analyses. J Benefit-Cost Anal. 2019;10(1):95-123. doi:10.1017/bca.2018.19

42. Aos S, Miller M, Drake E. Evidence-Based Public Policy Options to Reduce Future Prison Construction, Criminal Justice Costs, and Crime Rates. Washington State Institute for Public Policy; 2006. Accessed November 19, 2025. https://www.wsipp.wa.gov/ReportFile/952

43. Fowles R, Nyström S. Introduction to an Econometric Cost-Benefit Approach. Utah Commission on Criminal and Juvenile Justice; 2012. Accessed November 19, 2025. https://justice.utah.gov/wp-content/uploads/Utah-Cost-of-Crime-2012-Methods-Review-Cost.pdf

44. Schlueter M, Weber R, Bellas M, Morris W, Levery N, Greenewalt N. Criminal Justice Consensus Cost-Benefit Working Group. The Vermont Center For Justice Research; 2014. Accessed November 19, 2025. https://justiceresearch.dspacedirect.org/items/4d055dc4-92f6-44fa-bb12-3555af014a60

45. Expanded Homicide Data Table 8. FBI. Accessed April 30, 2025. https://ucr.fbi.gov/crime-in-the-u.s/2019/crime-in-the-u.s.-2019/tables/expanded-homicide-data-table-8.xls

46. Kottke-Weaver S. Justice and Employment Extracts Series. Bureau of Justice Statistics; 2019. Accessed November 19, 2025. https://bjs.ojp.gov/data-collection/justice-expenditure-and-employment-extracts-series

47. Boardman AE, ed. Cost-Benefit Analysis: Concepts and Practice. 4th ed. Prentice Hall; 2011.

48. Davis E, Goodison S. Law Enforcement Management and Administrative Statistics (LEMAS). Bureau of Justice Statistics; 2020. Accessed December 3, 2024. https://bjs.ojp.gov/data-collection/law-enforcement-management-and-administrative-statistics-lemas

49. Kyckelhahn T. Justice expenditure and employment extracts, 2012–Preliminary. Bur Justice Stat Febr. 2015;26. Accessed November 19, 2025. https://www.ojp.gov/library/publications/justice-expenditure-and-employment-extracts-2012-preliminary
